# Analgesia for total knee arthroplasty: a meta-analysis comparing local infiltration and femoral nerve block

**DOI:** 10.6061/clinics/2015(09)09

**Published:** 2015-09

**Authors:** ShuYa Mei, ShuQing Jin, ZhiXia Chen, XiBing Ding, Xiang Zhao, Quan Li

**Affiliations:** Tongji University School of Medicine, Shanghai East Hospital, Department of Anesthesiology, Shanghai, China.

**Keywords:** Total knee arthroplasty, Analgesia, Meta-analysis

## Abstract

Patients frequently experience postoperative pain after a total knee arthroplasty; such pain is always challenging to treat and may delay the patient's recovery. It is unclear whether local infiltration or a femoral nerve block offers a better analgesic effect after total knee arthroplasty.

We performed a systematic review and meta-analysis of randomized controlled trials to compare local infiltration with a femoral nerve block in patients who underwent a primary unilateral total knee arthroplasty. We searched Pubmed, EMBASE, and the Cochrane Library through December 2014. Two reviewers scanned abstracts and extracted data. The data collected included numeric rating scale values for pain at rest and pain upon movement and opioid consumption in the first 24 hours. Mean differences with 95% confidence intervals were calculated for each end point. A sensitivity analysis was conducted to evaluate potential sources of heterogeneity.

While the numeric rating scale values for pain upon movement (MD-0.62; 95%CI: -1.13 to -0.12; *p*=0.02) in the first 24 hours differed significantly between the patients who received local infiltration and those who received a femoral nerve block, there were no differences in the numeric rating scale results for pain at rest (MD-0.42; 95%CI:-1.32 to 0.47; *p=*0.35) or opioid consumption (MD 2.92; 95%CI:-1.32 to 7.16; *p=*0.18) in the first 24 hours.

Local infiltration and femoral nerve block showed no significant differences in pain intensity at rest or opioid consumption after total knee arthroplasty, but the femoral nerve block was associated with reduced pain upon movement.

## INTRODUCTION

Every year, approximately 130,000 patients receive knee replacements in the United States 1. Modern medical techniques offer substantial benefits for the increasing number of patients undergoing total knee arthroplasty 2,3. However, postoperative pain after total knee arthroplasty noticeably affects patients' quality of life. Clinical studies and hospital records have indicated that severe postoperative pain is associated with an increased risk of complications and that it slows the rehabilitation process, delays the improvement of persistent pain states, prolongs the length of hospital stay, and raises overall costs 4. Thus, greater attention should be paid to analgesia after total knee arthroplasty.

Pain management after total knee arthroplasty is a multipart strategy. It often includes intravenous opioids, epidural analgesia, or peripheral nerve blocks in combination with oral analgesics and cryotherapy 5. These treatments can have a number of side effects, such as nausea, emeses, excessive sedation, hypotension, inhibition of respiration, and urinary retention. A femoral nerve block is seen as the gold standard for total knee arthroplasty 6. However, local infiltration is becoming popular because it has fewer side effects and is easier to administer. Therefore, choosing effective analgesia methods is a topic of interest.

Using systematic review methods and meta-analysis, our objective is to synthesize evidence from randomized controlled trials to compare the effectiveness of local infiltration with that of a femoral nerve block for pain control in patients undergoing total knee arthroplasty.

## METHODS

### Search strategy and selection criteria

We searched articles in three electronic databases, Pubmed, EMBASE, and the Cochrane Library, for reports published between January 1, 2000, and January 26, 2015. The search terms included femoral nerve block, local infiltration, total knee arthroplasty, pain, analgesia, and randomized controlled trial. We removed duplicate articles that were located in more than one database. We included randomized controlled trials that compared the use of local infiltration with the use of a femoral nerve block for patients who underwent primary unilateral total knee arthroplasty. We excluded trials about bilateral total knee arthroplasty.

### Data collection and extraction

Two reviewers (Shuya Mei, Shuqing Jin) screened the relevant titles, abstracts, and full papers according to the previously mentioned criteria. If these two reviewers did not agree on a selection, a third reviewer (Quan Li) was consulted.

Information was extracted regarding study characteristics, participant characteristics, the anesthesia procedures common to randomized groups, intervention (methods for local infiltration and femoral nerve block and the concentration, volume, and dose of medicine), and analgesia assessment (numeric rating scale [NRS] values for pain at rest and upon movement and opioid consumption in the first 24 hours).

### Outcomes

Three aspects were assessed to compare the effectiveness of local infiltration and femoral nerve block for pain management in patients after total knee arthroplasty: 1. The NRS value for pain at rest in the first 24 hours; 2. The NRS value for pain upon movement in the first 24 hours; and 3. opioid consumption in the first 24 hours.

### Study quality

Possible biases were recorded in a Cochrane risk of bias table 7. Random sequence generation, allocation concealment, blinding of participants and personnel, blind outcome assessment, incomplete outcome data, selective reporting, and other sources of bias were measured with care. We classified the overall quality of each article as low, unclear or high risk of bias.

### Meta-analysis

Review Manager Software (Revman 5.0, Cochrane Collaboration, Oxford, United Kingdom) was used for the meta-analysis. The heterogeneity of the studies was tested using the I^2^ statistic and chi-squared tests. A fixed-effects model was used when the heterogeneity test did not reveal statistical significance (I^2^<50%, *p*>0.1). Otherwise, the random effects model was used. Three continuous variables were included in this meta-analysis (NRS value for pain at rest, NRS value for pain upon movement and opioid consumption in first 24 hours) and the mean difference (MD) and 95% confidence interval (95% CI) were determined. All tests of statistical significance were two-sided 8. A sensitivity analysis was performed using Stata 11.0 (StataCorp., College Station, TX, USA) to explore the effect of an individual study.

## RESULTS

### Search results

**Figure 1:**
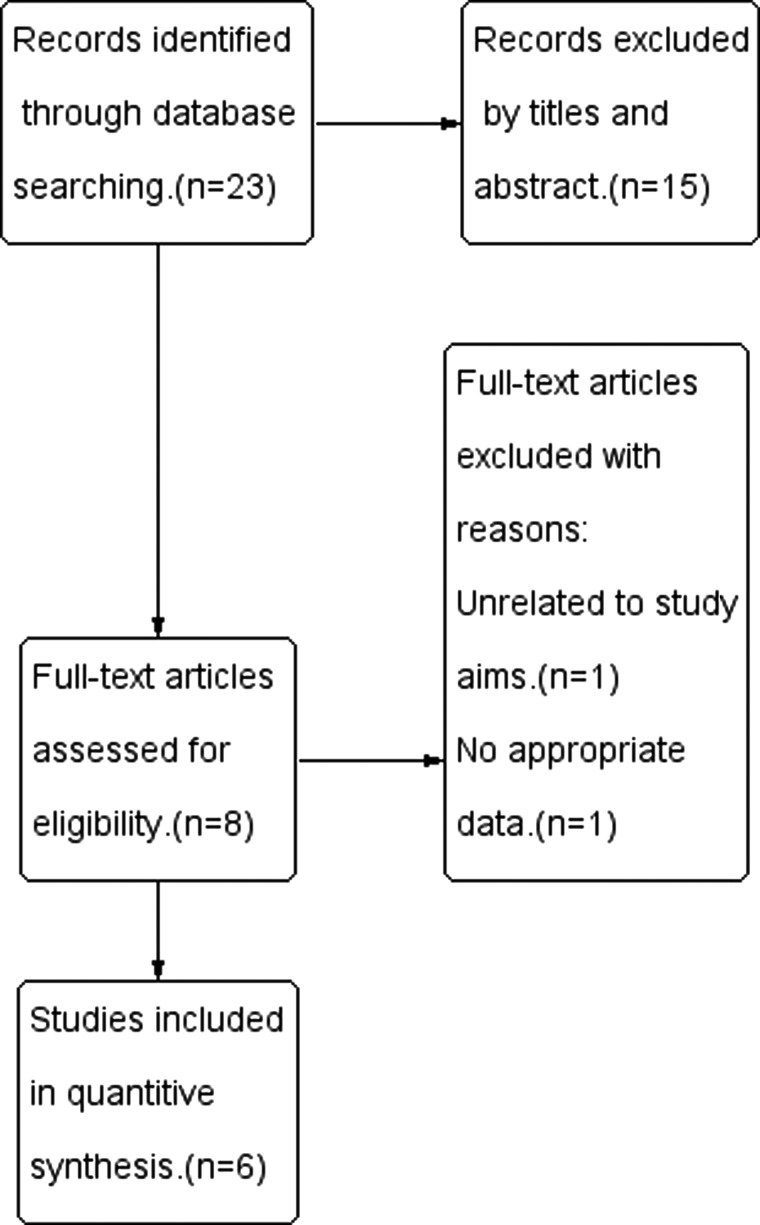
Flow chart of study selection.

Originally, 23 records were identified through the PubMed, EMBASE, and Cochrane Library databases. The full text of all articles except one 9 was available 5,, but only 8 of the articles 5, met the inclusion criteria. Among these, 1 article 24 was excluded because the study compared a femoral nerve block combined with periarticular anesthetic infiltration to local infiltration analgesia. One article 30 did not include data that could be extracted for our analyses. Ultimately, 6 articles were included in our meta-analysis. The specific electronic search strategy used is shown in Figure 1.

### Characteristics of the included trials

**Figure 2:**
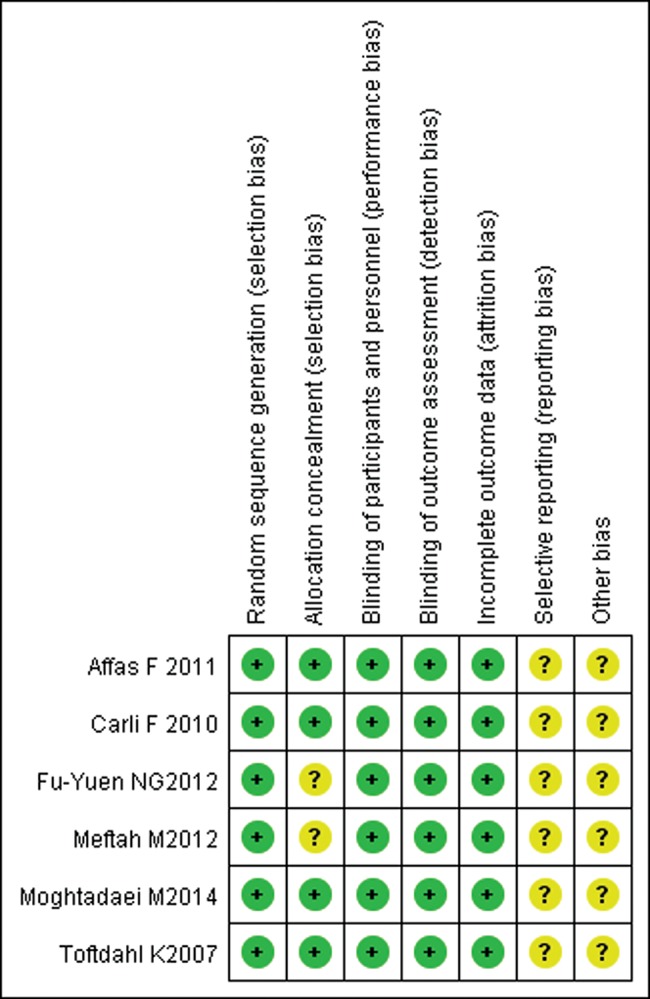
Risk of bias assessment for included studies.

Table 1 shows the detailed characteristics of the included trials, and Figure 2 shows risk of bias assessment results. All of the included randomized controlled trials had a low risk of bias and a moderate methodological quality. In all, 306 patients were included in the meta-analysis and were randomly allocated to receive either local infiltration or a femoral nerve block. The variety, concentration, volume, time, velocity and location of local anesthetics varied among the trials. Among the 6 included studies, all surgeries were primary unilateral total knee arthroplasties.

### Primary end points

**Figure 3:**
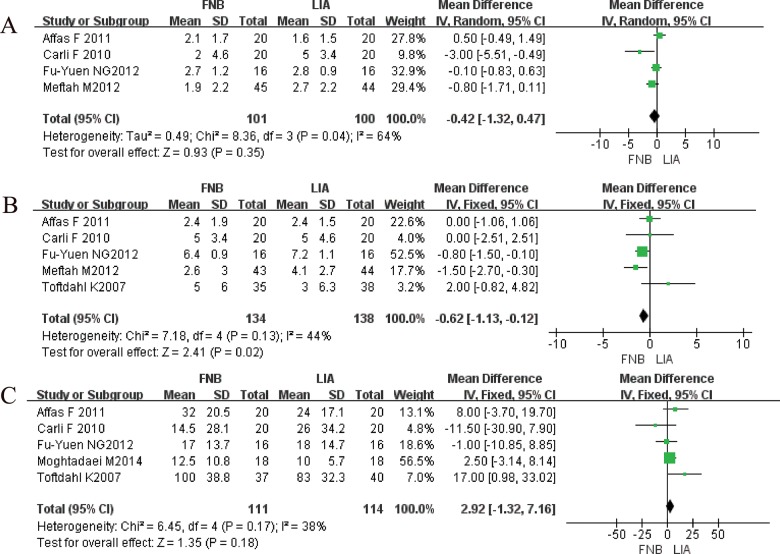
Meta-analysis results of local infiltration compared with femoral nerve block after total knee arthroplasty. (A) NRS value for pain at rest in the first 24 hours; (B) NRS value for pain upon movement in the first 24 hours; (C) opioid consumption (mg) in the first 24 hours.

The results of the NRS for pain at rest in the first 24 hours showed no differences between local infiltration and femoral nerve block (MD-0.42; 95%CI:-1.32 to 0.47; *p=*0.35; Figure 3A). However, significant differences were found in the NRS for pain upon movement in the first 24 hours (MD-0.62; 95%CI: -1.13 to -0.12; *p=*0.02; Figure 3B).

### Secondary end points

There were no differences in total opioid consumption in the first 24 hours between local infiltration and a femoral nerve block (MD 2.92; 95%CI:-1.32 to 7.16; *p=*0.18; Figure 3C).

### Sensitivity analysis

**Figure 4:**
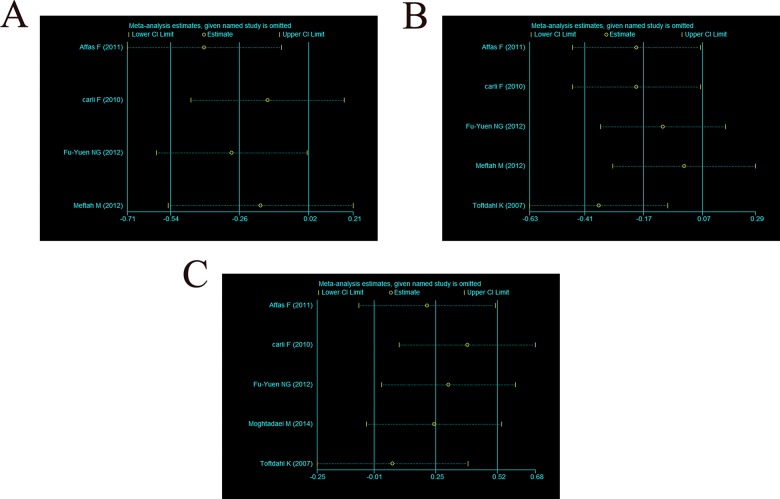
Sensitivity analysis of each end point. (A) NRS value for pain at rest in the first 24 hours; (B) NRS value for pain upon movement in the first 24 hours; (C) opioid consumption (mg) in the first 24 hours.

A sensitivity analysis of the three end points (NRS value for pain at rest, NRS value for pain upon movement and total opioid consumption in the first 24 hours) was conducted. No individual study significantly affected the heterogeneity results (Figure 4 A-C).

## DISCUSSION

We selected articles from various databases to obtain relatively fair and objective results. Ultimately, 6 out of 23 articles were included for analysis, most of which were randomized, controlled and double-blind experiments, except for two 27,28 whose design was not clear.

In comparing local infiltration and femoral nerve block for pain control after total knee arthroplasty, different studies drew different conclusions. Toftdahl K et al. 5 and Moghtadaei M et al. 29 proposed that local infiltration offered greater advantages for early analgesia after total knee arthroplasty. However, Carli F et al. 25 concluded that femoral nerve block was better for recovery from total knee arthroplasty. The remaining 3 studies 26-28 determined that both local infiltration analgesia and a femoral block provided good analgesia for total knee arthroplasty.

Our systematic review and meta-analysis provide a substantial evaluation of the effectiveness of local infiltration and femoral nerve block for pain control after total knee arthroplasty. Our data indicate that the two analgesic regimens offer a similar quality of pain relief at rest and are associated with similar levels of opioid consumption during the first 24 hours after total knee arthroplasty. The NRS value for pain upon movement was significantly lower in the femoral nerve block group.

Meftah M et al. 27 combined a femoral nerve block and patient-controlled epidural analgesia; they used bupivacaine for the block and infiltration, while others 5,25,26,28,29 used ropivacaine. Theodosiadis P et al. compared the efficacy and safety of ropivacaine and bupivacaine for a 3-in-1 block during total knee arthroplasty and found that ropivacaine and bupivacaine had similar analgesic effects 31. Muldoon T et al. determined that ropivacaine was associated with slightly higher pain scores than bupivacaine was, but the mean value of the difference might not reflect a significant clinical difference 32.

Patients in the study of Ng FY et al. 28 underwent general anesthesia, while patients in other studies 5,25-27, used spinal anesthesia. Liguori GA reported that the choice of anesthetic will affect not only the short-term success of post-operative analgesia but also the patient's ability to achieve certain physical therapy milestones 33.

Moghtadaei M et al. 29 employed a single-injection femoral nerve block, while others 5, employed continuous femoral nerve blocks. Francis et al. observed that a continuous femoral nerve block could extend the analgesic effect of a single-injection femoral nerve block after total knee arthroplasty 34. Chan EY et al. found that patients with continuous femoral nerve blocks had lower pain scores on movement at 24 h and consumed less opioids compared with patients who received single-injection femoral nerve blocks 35.

Although these three studies 27-29 had differences that may have affected the analgesic effects, only one article noted these differences. Therefore, subgroup analyses could not be performed. Our sensitivity analysis showed that no individual study significantly affected the heterogeneity results.

Our study has the following limitations: 1. Because of language and database limitations, only published full-text articles in English were included, and this resulted in small number of included studies; 2. No data about postoperative movement functions were extracted for analysis, so the assessment of analgesia for total knee arthroplasty was not comprehensive.

In summary, local infiltration provides analgesia comparable to that of a femoral nerve block for patients receiving total knee arthroplasty based on pain at rest and opioid consumption, but a femoral nerve block reduces pain upon movement.

## Figures and Tables

**Table 1:** Characteristics of eligible trials.

Author	Year of publication	Anesthesia	Number of patients	Femoral nerve block	Local infiltration
Toftdahl K	2007	spinal anesthesia	80	continuous femoral nerve block	local infiltration
Carli F	2010	spinal anesthesia	40	continuous femoral nerve block	local infiltration
Affas F	2011	spinal anesthesia	40	continuous femoral nerve block	local infiltration
Ng FY	2012	general anesthesia	16	continuous femoral nerve block	local infiltration
Meftah M	2012	spinal anesthesia	90	combine patient-controlled epidural analgesia and femoral nerve block	local infiltration
Moghtadaei M	2014	spinal anesthesia	40	single-injection femoral nerve block	local infiltration
